# Retinal imaging based glaucoma detection using modified pelican optimization based extreme learning machine

**DOI:** 10.1038/s41598-024-79710-7

**Published:** 2024-11-29

**Authors:** Debendra Muduli, Rani Kumari, Adnan Akhunzada, Korhan Cengiz, Santosh Kumar Sharma, Rakesh Ranjan Kumar, Dinesh Kumar Sah

**Affiliations:** 1 Department of Computer Science and Engineering, C.V. Raman Global University, Bhubaneswar, 751012 India; 2 Department of Computer Science, Birla Institute of Technology, Ranchi, Jharkhand 847226 India; 3 Department of Information Technology, Vi3, Image Analysis, Uppsala University, Uppsala, Sweden; 4 College of Computing and IT, Department of Data and Cybersecurity, University of Doha for Science and Technology, Doha, Qatar; 5 Department of Electrical-Electronics Engineering, Istinye University, 34010 Istanbul, Turkey; 6 Department of Computer Science and Engineering, Indian Institute of Technology, Dhanbad, 826001 India; 7 Division of Networked and Embedded Systems, Mälardalen University, 721 23 Västerås, Sweden

**Keywords:** ELM, FDCT-WRP, Glaucoma detection, IOP, LDA, MOD-POA, Engineering, Computer science

## Abstract

Glaucoma is defined as progressive optic neuropathy that damages the structural appearance of the optic nerve head and is characterized by permanent blindness. For mass fundus image-based glaucoma classification, an improved automated computer-aided diagnosis (CAD) model performing binary classification (glaucoma or healthy), allowing ophthalmologists to detect glaucoma disease correctly in less computational time. We proposed learning technique called fast discrete curvelet transform with wrapping (FDCT-WRP) to create feature set. This method is entitled extracting curve-like features and creating a feature set. The combined feature reduction techniques named as principal component analysis and linear discriminant analysis, have been applied to generate prominent features and decrease the feature vector dimension. Lastly, a newly improved learning algorithm encompasses a modified pelican optimization algorithm (MOD-POA) and an extreme learning machine (ELM) for classification tasks. In this MOD-POA+ELM algorithm, the modified pelican optimization algorithm (MOD-POA) has been utilized to optimize the parameters of ELM’s hidden neurons. The effectiveness has been evaluated using two standard datasets called G1020 and ORIGA with the $10 \times 5$-fold stratified cross-validation technique to ensure reliable evaluation. Our employed scheme achieved the best results for both datasets obtaining accuracy of 93.25% (G1020 dataset) and 96.75% (ORIGA dataset), respectively. Furthermore, we have utilized seven Explainable AI methodologies: Vanilla Gradients (VG), Guided Backpropagation (GBP ), Integrated Gradients ( IG), Guided Integrated Gradients (GIG), SmoothGrad, Gradient-weighted Class Activation Mapping (GCAM), and Guided Grad-CAM (GGCAM) for interpretability examination, aiding in the advancement of dependable and credible automation of healthcare detection of glaucoma.

## Introduction

Around the world, glaucoma is the second most prevalent disease, poses a significant threat to the optic nerve and can result in complete vision loss^1^. It has been observed that more than 60.5 million persons were affected under primary open-angle glaucoma (POAG) and primary angle closure glaucoma (PACG) globally in 2010, and it also predicted that can affect more than 112 million people by 2040^2^. It is a progressive disease which silently noticeable symptoms named “silent theft of sight”. Hence, early detection and screening of glaucoma is essential for timely treatment. Tonometry is a technique used to measure the fluid pressure in the eyes, known as intraocular pressure (IOP), as a part of the assessment for detecting glaucoma. One limitation is that all healthcare centres are not equipped to perform visual field tests. The Optic Nerve Hypoplasia (ONH) assessment has been the more prominent to find clinically. Researchers have also investigated adaptive optics, which can correct for aberrations in the eye and provide even higher-resolution images. Finally, there has been growing interest in developing new biomarkers for glaucoma. Biomarkers are measurable indicators of disease that can be detected in body fluids or tissues. Several authors have identified potential biomarkers for glaucoma, including changes in the levels of specific proteins and metabolites in the eye. Regular eye examinations are essential for early detection and treatment of glaucoma, as vision loss from the disease is irreversible. Recent advances in glaucoma detection have focused on developing new technologies and methods, such as artificial intelligence and machine learning algorithms, which can analyse retinal images and predict the risk of developing glaucoma^3^. The manual expansion of detecting fundus images is costly, complicated and computationally time-consuming for ophthalmologists. Therefore, if the optic nerve in the back of the eye, gets injured and is not treated correctly, it becomes extremely challenging to identify the problem Consequently, to assist the ophthalmologist in accurately identifying the fundus image, an enhanced CAD model that relies on automated fundus image detection is utilised for this purpose. It enhances productivity and increases the outcomes of detecting several types of fundus images^4,5^. Different CAD models have been deployed on neural networks to detect glaucoma. Because of their ability to simplify and provide global approximations, neural networks are employed in numerous research studies to tackle classification and regression tasks. But, the basic limitation of neural network models like overfitting, slow convergence, and local minima. It is an omnipotence and prominent ML classifier named as ELM^6,7^, that based on the correlation between the learning mechanisms within adaptive layered networks and the procedure of modeling data through intricate, high-dimensional surfaces^8^. A specific type of adaptive network is recognised that explicitly defines the interjection method, which obtained greeted speeds and enhanced generalisation outcomes. Namely,ELM conventional neural networks, training has not necessitate iterations. But instead, the input-to-hidden-layer weights are randomly generated, and the output weights are computed using an analytical approach. To mitigate such issues, the neural network is trained using a potent learning technique called extreme learning machine (ELM)^7^, radial basis function network (RBFN)^8^, offers enhanced speed and delivers superior performance outcomes compared to alternative methods. The input weights are non-linearly estimated by transforming the input layer to the hidden layer within the ELM framework.Therefore, in order to ascertain the weights connecting the hidden and output layers, excluding parameter tuning, an explanatory approach known as Moore-Penrose (MP) is employed. Due to faster learning speed, ELM has been widely employed across various research domains, including the classification of breast tumours^9–12^, diabetes detection^13,14^, etc. Manghnani et al.^15^ proposed a new CAD model based on an adaptive approach for glaucoma detection using bidimensional empirical mode decomposition (BDEMD) from retinal images. Then, Adaptive bi-dimensional intrinsic mode functions (BDIMFs) are obtained using very simple steps, from pre-processed and coloured decomposed images then normalized and classified by a support vector machine (SVM) with its different kernel functions. In^16^, the authors have presented a novel model on glaucoma prediction using bi-dimensional intrinsic model functions(BDIMFs)based feature fusion then normalized and SVM has been used for classification with 10-fold cross-validation(FCV). In^17^, the authors employed a new deep learning model with different potential models namely Inceptionv3, ResNet50, VGG19, Xception and custom Convolutional neural network(CCNN). VGG19 achieved better classification accuracy than other deep-learning models for glaucoma detection. Kikar et al.^18^ presented a new CAD model based on second-stage quasi-bivariate vibrational model decomposition (SS-QB-VDM) based sub-band images from fundus images for glaucoma detection. Then, in the second stage decomposed SBIs are fine with no mode mix problems and SVM is utilized for glaucoma classification. Kikar et al.^19^ have used a new CAD model for glaucoma detection using image channels(ICs)and discrete wavelet transform(DWT) for feature extraction. The robust features are selected and fed to the least square support vector machine (LS-SVM) for classification which achieves an accuracy of 84.95% than other traditional models. Agrawal et al.^20^ presented a novel model and more accurate method for automated glaucoma detection using quasi-bivariate variational mode decomposition (QB-VMD) from digital fundus images. Based on better detection results they have used seventy features extracted from QB-VMD SBIs. Extracted features are normalised and selected using the ReliefF method. Selected features are then fed to singular value decomposition to reduce their dimensionality. Finally, the reduced features are classified using the least square support vector machine classifier for glaucoma detection. Kikar et al.^21^ used a CAD model that employed the concatenation approach which is the combination of all features obtained using DWT and EWT and their combination. Extracted features from each of DWT, EWT, DWTEWT and EWTDWT are concatenated. Concatenated features are normalised, ranked and fed to singular value decomposition to find robust features. Fourteen robust features are used by the support vector machine classifier to achieve better classification results. Kikar et al.^22^ used a novel fully variational and adaptive computer-based glaucoma detection using compact variational mode decomposition (CVMD) from fundus images. Then, Efficient sub-band images having narrow Fourier bandwidth, and clear and sharp boundaries are obtained using CVMD which gives a robust estimate of features. Texture features capture subtle variation and give more information therefore these features are extracted from efficient subband images and help to increase the glaucoma detection accuracy. Extracted features are normalized and classified using a support vector machine classifier. Explainable artificial intelligence (XAI) is crucial in the medical field as it aids in enhancing the understanding of deep learning models^23^. Diagnostic tasks are prone to errors due to subjective judgments, and explainability can make these systems more transparent, leading to better evaluation and addressing issues related to accountability, fairness, and ethics^24^ . As a result, XAI holds significant practical value in medical imaging. By using XAI, healthcare professionals can better grasp the reasoning behind medical conclusions and more effectively utilize deep learning technologies^25^. The demand for explainable approaches in medical imaging is growing, and in the European Union, XAI in medical contexts is now mandated under the General Data Protection Regulation (GDPR)^26^.

This study aims to develop an automated computer-aided detection (CAD) model designed explicitly for classifying fundus images. Our deployed scheme has been applied to ELM, an excellent learning approach used for glaucoma detection with a modified pelican optimization algorithm (MOD-POA) to address the constraints inherent in ELM, we fine-tuned the input weight and bias of the model to enhance optimization. The prime contributions are listed below:

- To extract features, specifically a collection of curves, from glaucoma images, the fast discrete curvelet transform with wrapping ( FDCT-WRP) schemes has been employed for capturing 2D singularities features and integrated into the methodology.
- To enhance traditional learning methods and achieve faster learning speed and superior performance, an improved version of a single hidden layer feed-forward network (SLFN) called extreme learning machine (ELM) is employed.
- The hybridized approach combining the modified pelican optimization algorithm and ELM (MOD-POA+ELM) has been implemented to enhance the learning process by effectively disregarding unnecessary hidden nodes, avoiding local minima, and optimizing the speed of response for testing data.
- We present a glaucoma classification task that utilizes re-parameterization. This model offers simplicity and efficiency, effectively capturing both cup-to-disc ratio (CDR) information, while also addressing classification and explainability tasks based on seven XAI methods.

## Related works

During the past decade, numerous proposed CAD models have introduced to classify glaucoma using fundus images. Most CAD models have three distinct components: feature extraction, selection, classification. The classification of fundus images heavily relies on detection of glaucoma plays crucial roles of extraction of features and dimensional reduction, significantly impacting the classification process. The primary objective of ONH assessments is to perform binary classification distinguishing between individuals with glaucoma and healthy. It is essential to accurately segment the optic disc and cup to detect the cup-to-disc ratio (CDR) precisely. Based on given the time-consuming nature of manually segmenting the optic disc and cup, the cup-to-disc ratio (CDR) is often assessed by comparing the vertical diameter of the cup to that of the disc. From majority of CAD models utilize various features, such as spatial domain, frequency domain, textual domain, statistical domain, and multi-resolution behavior, to classify glaucoma fundus images^27,28^. There are three main categories of techniques for optic disc segmentation: template-based methods, deformable model-based methods, and pixel classification-based methods^29,30^.

The utilization of multi-resolution techniques such as wavelet and curvelet offers a proficient and compact way to represent images and wavelet characteristics have been employed in various CAD models to address classification challenges^31^. The method proposed by Agboola^32^ employs wavelet coefficients to achieve multi-resolution glaucoma analysis, resulting in enhanced outcomes. They explored a combination of statistical characteristics along with a binary tree as a classification tool in their approach. Akhras et al.^33^ introduced an innovative framework, the team has unveiled an advanced approach leveraging machine learning-based classification to potentially revolutionize the early and automated diagnosis of glaucoma. In this model, support vector machines and finely tuned artificial neural networks serve as the key components, functioning as a robust classifier. Usman et al.^34^ have introduced a CAD system designed to incorporate multiple techniques such as Fourier domain OCT systems (FD-OCT), spectral domain OCT (SD-OCT), and swept-source OCT (SS-OCT). They have utilized several methods involving a laser that sweeps across different frequencies and a high-capacity balanced photo-detector to capture images. Khan et al.^35^ have proposed a new methodology to classify automated fundus images by deploying a non-gaussian bivariate distribution function based on a feature selection algorithm and SVM as a classifier with several kernel functionalities. Acharya et al.^36^ have presented an innovative automated method for detecting glaucoma is presented in this study. The approach incorporates adaptive histogram equalization to transform RGB images into grayscale. Convolution is then applied using diverse filter banks, including Leung-Malik (LM), Schmid (S), and higher-response (MR8, MR4) filters.. They utilized six features extracted from the LM filter bank and applied the K-nearest neighbours (KNN) as a classifier. Dey et al.^37^ presented a CAD model, which utilizes machine learning techniques based on statistical feature extraction techniques, particularly texture features are derived from fundus images through the analysis of the Grey-Level Run Length Matrix (GLRLM) and Grey-Level Co-occurrence Matrix (GLCM). The classification task is executed through the application of a Support Vector Machine (SVM) classifier. In^38^, introduced innovative computer-aided design (CAD) methods leveraging the accelerated robust feature (SURF) and histogram of oriented gradients (HOG) characteristics. Such scheme incorporates an advanced hybrid optimization technique in conjunction with SVM for performing classification tasks.

In^39^ the authors used an automated diagnostic system employing a model incorporating histogram of oriented gradients (HOG) in conjunction with a feed-forward neural network (FFNN) In^20^, have implemented an innovative approach that combines quasi-bivariate variational mode decomposition (QB-VMD) with the ReliefF algorithm for efficient feature selection. As a result, we chose to use the highly efficient least squares support vector machine (LS-SVM) for the classification task. In^40^, the authors have employed an alternative CAD model, we applied the flexible analytic wavelet transform (FAWT) to extract a range of entropies and fractal dimension (FD) characteristics. To reduce feature dimensions, we utilized linear discriminant analysis (LDA) and implemented a least-squares support vector machine (LS-SVM) as a classifier. In^41^, the authors employed novel CAD model employs DWT and HOG features, with subsequent classification performed using an ELM classifier. Balasubramanian et al.^42^, introduced a ML technique which leverages correlation features selected through a bio-inspired algorithm. Additionally, the classification task is performed using a KELM (Kernel Extreme Learning Machine) with optimization based on salp-swarm methodology. Morales et al.^43^ havesuggested a novel Computer-Aided Design (CAD) model for optic disc extraction employing mathematical morphology and principal component analysis (PCA). This approach enhances the grayscale image quality compared to the original RGB. For OD segmentation, they have focused on different mathematical morphology like centroid calculation, stochastic watershed, and region discrimination; post-prepossessing has been used as circulation approximation. Huang et al.^44^ have presented a CAD model that builds upon ELM and can be expanded to include the radial basis function (RBF) enables the random generation of centers and impact widths for RBF kernels, with the subsequent iterative calculation of output weights through analytical methods.

In^21^, introduced a novel method that employs extraction of features methods like discrete wavelet transforms (DWTs) and empirical wavelet transforms (EWTs) have been utilized to decompose images. They combined all the features obtained through DWT and EWT. They subsequently normalized the concatenated set of features, ranked which fed to singular values decomposition and LS-SVM algorithm used for classiffication. Maheshwari et al.^45^ have introduced a novel method for diagnosing glaucoma, utilizing empirical wavelet transform (EWT), and the t-value feature selection algorithm is employed to rank the features. They applied the least squares support vector machine (LS-SVM) classifier to perform classification using radial basis function, market wavelet, and Mexican-hat wavelet kernels. In^46^, presented a CAD method on linear discriminant analysis (LDA) and artificial neural network (ANN) techniques have been applied to improve the differentiation between glaucomatous and normal eyes within the Taiwanese Chinese population, taking into account the measurement of retinal nerve fiber layer thickness^47^. They have used ANN, LDF, and NFI methods to demonstrate equal diagnosis power in glaucoma detection. Huang et al.^48^ use computational intelligence approches, i.e., neural network, support vector machine (SVM) as a classifier, and ELM works for generalized single-hidden layer feed-forward networks (SLFNs). In^49^, used an innovative learning algorithm known as ELM, accompanied by a hybrid learning approach for the selection of input weights. Additionally, the Moore-Penrose (MP) generalized inverse is employed to analytically determine the output weights in our proposed methodology. Dua et al.^50^ have used The demonstrated discriminatory capability of employing wavelet filters such as Daubechies (db3), eyelets (sym3), and biorthogonal (bio3.3, bio3.5, and bio3.7) for feature extraction, in conjunction with a Support Vector Machine (SVM) classifier, has been showcased.

From the literature, it has come to notice that a majority of CAD models extract features from two-dimensional wavelets, such as DWT, SWT, EWT, and similar methods. The fundus images have subjected to various levels of segregation and subsequently transformed into one-dimensional representations for experimental purposes. Hence, the observation indicates that DWT is inadequate for extracting the curve sample features in glaucoma images. So, SLFN, SVM, and ELM are commonly utilized in various CAD models for classification purposes; this results in more parameters being tuned, leading to a longer computational time.

In this article, we have introduced a model which effectively captures two-dimensional singularities; a fast discrete curvelet transform with wrapping (FDCT-WRP) method has been employed for extraction of features that capture 2D singularities,method of dimensionality reduction, “PCA+LDA”,used for choose pertinent features. Due to fundus digital image detection, a modified extreme learning machine (ELM) called MOD-POA+ELM utilized on POA (pelican optimization algorithm) to enhance the learning process of the traditional ELM algorithm^51^. It’s merits of ELM are reducing the number of hidden nodes, avoiding local minima, list of remote nodes. Our implemented model has evaluated using two widely used datasets, G1020^52^ and ORIGA^53^. Digital database for screening of fundus images in G1020 and ORIGA, the performance analysis with traditional models to assess the efficacy of the implemented model, and validation has been performed.

Additionally, we introduce seven existing explainable artificial intelligence (XAI) methods integrated with our classification approach namely Vanilla Gradients (VG)^54^, Guided Backpropagation (GBP)^55^, Integrated Gradients(IG)^54^, Guided Integrated Gradients( GIG)^56^, SmoothGrad^57^, (Gradient-weighted Class Activation Mapping (GCAM)^58^, Guided Grad-CAM ( GGCAM)^24^. Our goal is to demonstrate a glaucoma identification method that outperforms previous studies, while incorporating explainability features to enhance trust in the system. The proposed model has been implemented as a web application, allowing practitioners to use it as a support tool.

## Methods

**Fig. 1:**
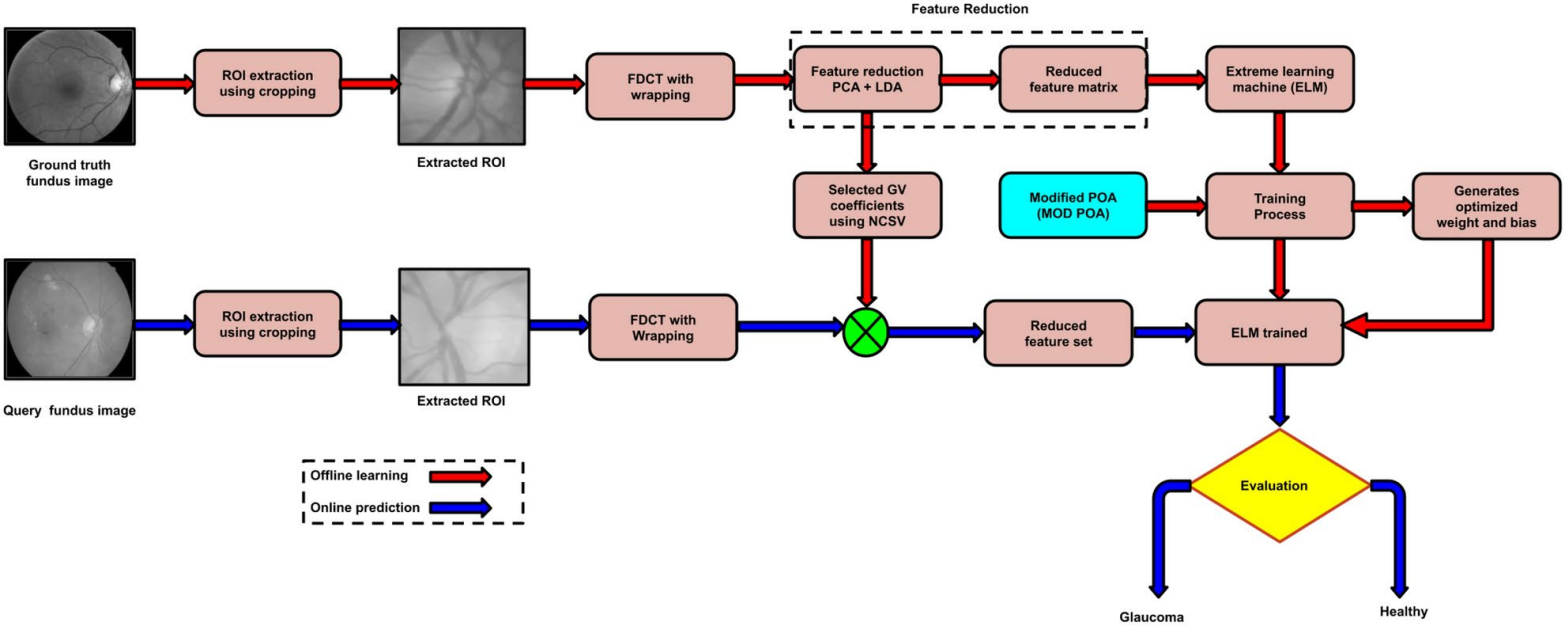
Comprehensive overview of the Proposed model block diagram.

The deployed scheme comprises four key components: pre-processing, extraction of features, feature reduction, and classification. In the preprocessing stage, relevant features are obtained by applying the fast discrete curvelet transform with wrapping (FDCT-WRP) for the extraction of the region of interest (ROI) from fundus images. Then, removing relevant features is accomplished sequentially by employing PCA, and LDA. The relevant features extracted from the preprocessing stage are ultimately fed into the optimized modified pelican optimization algorithm and extreme learning machine (MOD-POA+ELM) for classification (Fig. 1).

**Fig. 2:**
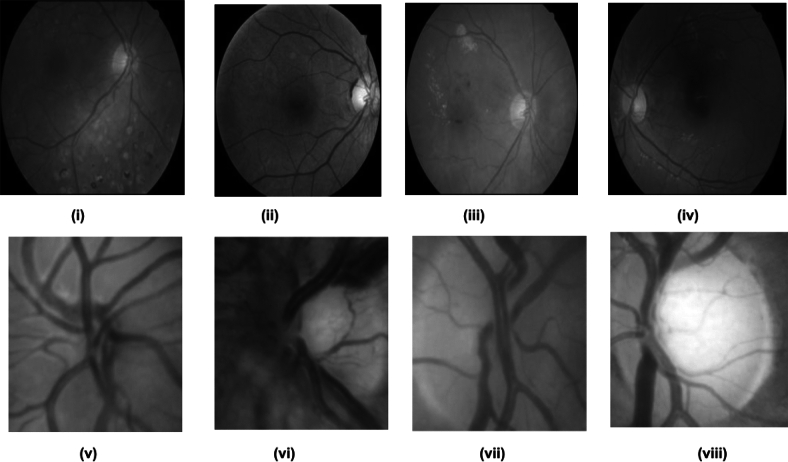
Some digital fundus images and their respective ROIs of Glaucoma datasets ((i) to (iv)) original images and ((v) to (viii)) their corresponding ROIs (Source of images are G1020^52^ and ORIGA^53^).

In our implemented scheme, the glaucoma data sets have been split into a training set and a testing set using a specific 60:40 ratio and testing better classification results. In our proposed work, we presented two online repositories, G1020^52^ and ORIGA images^53^. Usually, fundus images contain several unwanted components, namely noise, artifacts, test information, etc., during acquisition. These abnormalities continue to spread to the extracted features if they are obtained without eliminating such artifacts. Therefore, standard techniques for image preprocessing, such as enhancement and noise reduction methods, are utilized in all images. Today, the enormous amount of fundus images obtained primarily stored locally, due to exploitation of the valuable embedded fundus images, could be more efficient. Our model has assessed the cropping procedure to isolate the relevant region of interest (ROI). To streamline processing time, manually identified regions of interest (ROIs) are employed^50^ extracted using cropping techniques for both datasets, namely G1020 and ORIGA. The ophthalmologists delineated the values of cup and disc ratio in numerous images. Our proposed approach is centered around cropped images with a specified size of 128 $\times$ 128 as shown in Fig. 2. The Comprehensive overview of the proposed model illustrate the overall workflow and depicts the central theme shown in Fig. 1.

### Feature extraction utilizing FDCT-WRP

The widespread use of wavelet transform is attributed to its multi-resolution characteristics, allowing for extracting time-frequency localized information from images. Nevertheless, conventional wavelet methods need help in detecting two-dimensional singularities, such as lines and curves, in contrast to the capabilities of ridgelet and curvelet transforms. The Ridgelet transform can extract diverse features such as lines and orientations, but it needs more efficient handling curve singularities within an image^59,60^. Addressing this limitation, the initial generation of the curvelet transform has introduced by Candes and Donoho, utilizing a multi-scale ridgelet approach^61^. This variant offers enhanced attributes, including increased directional sensitivity, multi-resolution adaptability, anisotropy, and improved localization. An advanced iteration of the curvelet, known as the second generation curvelet transform, has been deployed on^62^ to address the limitations of the initial generation of curvelet transforms, which encompassed issues such as ambiguous identification of ridgelets and excessive computational burden^63^. In the following, we elucidate the essential mathematical derivation of discrete curvelet transforms. In signal $S_i$, the curvelet transform $C_U$($p_c$, $q_c$, $r_c$) has explained as the inner product of $S_i$, $\tau _{{p_c},{q_c},{r_c}}$ that is

1 $$\begin{aligned} C_U(p_c, q_c, r_c)=<S_i, \tau _{{p_c},{q_c},{r_c}}> \end{aligned}$$

Where, $\tau _{{p_c},{q_c},{r_c}}$ shows the primary function of the curvelet, $p_c$, $q_c$, and $r_c$ shows the list of the parameters such as scale, position, and orientation as needed. In the process of curvelet transformation, the image is divided into multiple windows at different scales and orientations. The discrete form of a curvelet transform is obtained based on the input image $\int [a^1,b^1]$ with $\ 0 \leqslant x^1, y^1 < m$ is expressed as

2 $$\begin{aligned} {C_U ^{{\hat{D}}} \left( p_c,q_c,r_c\right) = \sum \limits _{0 \le a^1, b^1 < n} S\left[ a^1,b^1\right] \overline{\tau ^{{\hat{D}}}_{p_c, q_c, r_c}[a^1,b^1]}} \end{aligned}$$

Hence, $\tau ^{{\hat{D}}}_{ p_c, q_c, r_c}$ The provided image exhibits an automated digital curvelet waveform. The progression of the $2^{nd}$ generation curvelet transform involves two distinct processes: wrapping and the unequally spaced first Fourier transform (USFFT). Based on the first-generation curvelet, these methods are characterized by simplicity, being the first and having the least redundancy. Hence, the FDCT - WRP method is more straightforward and prominent than USFFT in two procedures. Taking into account that, in our research, for feature extraction. We have utilized wrapping plans to construct by initial discrete curvelet transform known as FDCT - WRP. Consider the following list of steps to be considered for the implementation of FDCT - WRP.

- Perform a 2D Fast Fourier Transform (FFT) calculation and obtain its coefficients ($V[c^1,d^1]$) from every image.
- Generate a discrete window is created for each scale and angle, providing localization within the given context within the Fourier domain (${\hat{X}}_{p_c,q_c}[c^1,d^1]$) and evaluate the product (${\hat{X}}_{p_c,q_c}[c^1,d^1]$,$V[c^1,d^1]$)
- By utilizing the re-indexing method, the data undergoes processing as it is wrapped around the origin (${\hat{V}}_{p_c,q_c}[c^1,d^1]$)
- To define the discrete curvelet coefficients $C_U{\hat{D}}_{p_c,q_c,r_c}$, by conducting the reverse 2D FFT on ${\hat{V}}_{p_c,q_c}$.

The fast curvelet transform incorporates the coefficient with a wrapping procedure at each scale and orientation, similar to $p_c, q_c$, to create the feature vectors. To determine the list of rankings, the size of the image has taken into consideration $m^r, m^c$ is computed as:

3 $$\begin{aligned} {\hat{s}} = \lceil {\log _{2}{(min(m^r, m^c)}-3}\rceil \end{aligned}$$

Here, each image dimension is 128 $\times$ 128, it’s value ${{\hat{s}}}$ value is 4, which means every image is sub-divided the curvelet transforms into four levels. Exclude the first and last scales and consider the remaining scales (2,3) of several substandard information. At angles $\alpha$ and $\alpha +\pi$, the curvelet creates similar coefficients at every scale. Hence, at every scale, consequently, the coefficients in the feature vectors are of significant magnitude; further reduction is required to choose the essential features, necessitating additional feature reduction.

Dimensionality reduction serves as a technique in machine learning and data analysis, aimed at diminishing the multitude of input features or variables within a dataset. The fundamental objective is to streamline the dataset, retaining essential information while minimizing complexity.

Principal Component Analysis (PCA) and Linear Discriminant Analysis (LDA) are techniques utilized for feature dimensionality reduction in machine learning. PCA strives to discover a reduced-dimensional representation of the data while retaining the maximum possible variability from the original dataset. It identifies the primary directions of variation within the data and maps the data onto a reduced subspace defined by these identified directions. This specific subspace, known as the primary subspace, is formed by the principal components, which are the eigenvectors derived from the covariance matrix of the dataset. Conversely, LDA is a supervised method designed to discover a reduced-dimensional representation of the data, with the objective of optimizing the distinction between various classes. t operates by mapping the data onto a subspace where the variance between classes is maximized and the variance within each class is minimized. The resulting subspace is chosen to maximize Fisher’s linear discriminant criterion. This approach is practical in various applications, such as face recognition and image classification. Combining the two techniques and employing this method makes it feasible to decrease the dimensionality of the data while simultaneously optimizing the discrimination between different classes, leading to better performance on classification tasks.

The efficacy of Linear Discriminant Analysis (LDA) may be enhanced in scenarios involving with high-dimensional features and constrained sample sizes, the scatter matrix serves as a representation of ($S_m$) is seldom remarkable^64^. Such a problem is called a small sample size (SSS). To form $S_m$ denoted by matrix of non-singular, lowest ${\hat{D}} + C$ (so, ${\hat{D}}$ defines the size of the feature matrix and enumerates the classes as *C* ) list of technically inapplicable features required^65^. Our deployed scheme incorporates a fusion reduction technique known as $PCA-LDA$ In the course of our implemented efforts to tackle this issue. At first, ${\hat{DE}}$ specified as diminution of feature vector data PCA on *MA*-hyper-parameteres afer that *LD*- specified as Linear Discriminant Analysis (LDA) feature parameter and, then final parameters is $LD< MA <{\hat{DE}}$. We arrange the estimated eigenvalues of the elements in descending order to identify the most critical characteristics. So, *NCSOV*, method is provided for calculating the characteristics of every feature normalization sum of cumulative variance. Then, $i^{th}$ parameters *NCSOV* have been evaluated by:

4 $$\begin{aligned} NCSOV(i)=\frac{\sum _{y=1}^{i}{\beta {(y)}}}{\sum _{y=1}^{{\hat{DE}}}{\beta {(u)}}}: here,1\leqslant i\leqslant {\hat{DE}} \end{aligned}$$

Now, $\beta {(y)}$ specified as eigenvalue of $y^{th}$ feature, then, parameters of feature matrix as ${\hat{DE}}$. Lastly, our simulation have considered the threshold value manually and selected features as relevant when *NCSOV* value enhance the threshold value. The initial LD number of eigenvectors meeting the specified condition is identified as the resultant reduction feature for the classification task.

### MOD-POA+ELM-based classification

This segment is structured into three parts to enhance comprehension: the traditional extreme learning machine (ELM), the pelican optimization algorithm (POA), and the integrated novel classification approach known as MOD-POA+ELM. In current decades, single-hidden layer feed-forward neural networks (SLFNs) have found extensive application in approximating continuous functions and identifying separate regions. Learning algorithms based on gradients, like The Levenberg-Marquardt (LM) and back-propagation (BP) algorithms have been widely utilized for the training of these Single-Layer Feed-forward Networks (SLFNs). However, several challenges associated with this learning method are observed, including slow training speed, susceptibility to getting stuck in local minima, overfitting, and extensive iterations due to inadequate learning steps^6,66^. A recently developed learning technique, called the extreme learning machine (ELM), effectively addresses these inherent in gradient-based learning approaches. ELM surpasses these limitations and demonstrates the capability to excel in identifying high-performance patterns and resolving regression functions^67^. In ELM, The values of parameters such as input weights and hidden biases for the hidden node are assigned randomly. In contrast, the output weights of the ELM are determined through a straightforward inverse operation that involves the output matrix of the hidden layer. The mathematical derivation of the ELM approach is elaborated below.

We used $\mathbb {{\hat{N}}}$ as set of distinct training sample $(r_i,s_i)$, where, $r_i=\left[ r_{i1},x_{i2},\ldots ,r_{iL}\right] ^T \in R^L$ and $s_i=[s_{i1},s{i2},\ldots ,$ $h_{iC}]^T \in R^C$. The activation functions $\delta (.)$, number of hidden nodes and *SLFNs* consist of having $s_{hidden}$ and are listed as:

5 $$\begin{aligned} \sum \limits _{i=1}^{t_s}wve^o_i \delta (r_j)=\sum \limits _{i=1}^{t_s}wve^o_i \delta (wve^s_i \cdot r_j + {\hat{b}}_i)=o_j, \ \ j=1,2,\ldots ,\mathbb {{\hat{N}}} \end{aligned}$$

Here, the weight vector is $wve^s_i=\left[ wt^s_{i1},wve^s_{i2},\ldots ,wt^s_{il}\right] ^T$ which specifies a connection based on hidden neuron ($i^{th}$) and the input neurons. The interpretation likely revolves around the weight vector of the concealed neuron $wt^o_i=\left[ wve^o_{i1},wve^o_{i2},\ldots ,wve^o_{iC}\right] ^T$. Finally, the bias of the hidden neuron $i^{th}$ denoted as ${\hat{b}}_i$. The projected significance of SLFNs in $\mathbb {{\hat{R}}}$ is error-free.

6 $$\begin{aligned} \sum \limits _{i=1}^{t_q}wve^o_i \delta (wve^q_i \cdot p_j + {\hat{b}}_i)=q_j, \ \ j=1,2,\ldots ,\mathbb {{\hat{N}}} \end{aligned}$$

The explanation for the vector format represented by Equation 6 is provided below::

7 $$\begin{aligned} {\hat{\textbf{HI}}}wve^o={\hat{\textbf{T}}} \end{aligned}$$

Here, $${\hat{\textbf{HI}}}(wve^h_1,wve^h_2,\ldots ,wve^h_{ve_q},{\hat{b}}_1,{\hat{b}}_2,\ldots ,{\hat{b}}_{v_h},r_1,r_2,\ldots ,r_\mathbb {{\hat{N}}})$$

The vector resulting from the hidden layer can be acquired by ${\hat{\textbf{HI}}}$. After, its resultant weight $wve^o$The assessment involved examining the outcome of the lowest norm least squares (NLS) to determine as:

8 $$\begin{aligned} \hat{wve^o}={\hat{\textbf{HI}}}^\dagger {\hat{\textbf{T}}} \end{aligned}$$

For (Eq. 8), ${\hat{\textbf{HI}}}^\dagger$ viewed as Moore–Penrose (MP) matrix Inverse using simplified form is ${\hat{\textbf{HI}}}$, Streamline the extreme learning machine through simplification. The latest solution boasts the lowest norm value compared to all other Nonlinear Least Squares (NLS) solutions. Consequently, ELM exhibits superior speed compared to the majority of traditional learning algorithms, requiring fewer iterations for hyperparameter tuning.

### Proposed modifed-pelican optimization algorithm (MOD-POA)

The developed MOD-POA has been used to choose optimal features; and the optimization process has enhance the classification accuracy of the deployed glaucoma fundus image detection. Pelican optimization algorithm consists of better competitive results and an effective balance between exploration and exploitation comparing algorithms for competitiveness, ensuring optimal resolutions for optimization challenges^68^. Therefore, challenges arise when assessing the POA’s performance in real-life situations. So it is required to develop an enhanced MOD-POA method. It helps to reduce the convergence rate, and also it attains a greater accuracy rate. It solves constraint optimization problems and can deploy in worldwide applications. It deployed to a large number of datasets. In the improved method of MOD-POA, the prey position is updated by Eq. 9.

9 $$\begin{aligned} loc=\frac{BE + WO}{2} \end{aligned}$$

Here, the loc denotes the prey’s location, and the terms BE and WO represent the parameter optimisation’s best and worst index values. **Initialization:** It is considered that there are U be the number of pelicans in V dimensional space, the location of an $i^{th}$ pelican in V dimensional space is expressed as [$Ri= ri_1, ri_2, ri_3, \dots ri_V$]. The location $r_i$ of *U* the pelicans is represented in Eq. 10

10 $$\begin{aligned} r_{i,j}= & {loc}_{bj} + ra \cdot (u_{bj} - l_{bj}), \quad i=1,2,3,\dots , U, \quad j=1,2,3,\dots , V \end{aligned}$$

11 $$\begin{aligned} {\textbf{R}}= & \left[ {\begin{array}{c} Ri_1 \\ Ri_2 \\ \vdots \\ Ri_i \\ \vdots \\ Ri_u \\ \end{array}}\right] =\left[ {\begin{matrix} ri_11 & ri_i2 & \ldots & ri_1u & \ldots & ri_1u \\ ri_12 & ri_22 & \ldots & ri_2u & \ldots & ri_2u\\ \ldots & \ldots & \ldots & \ldots & \ldots & \ldots \\ ri_i1 & ri_i2 & \ldots & ri_iu & \ldots & ri_iu\\ \ldots & \ldots & \ldots & \ldots & \ldots & \ldots \\ ri_u1 & ri_u2 & \ldots & ri_uv & \vdots & ri_vu\\ \end{matrix}}\right] \end{aligned}$$

Where, $R^i$ is specified as the population matrix of an $i^{th}$ pelican, in POA, every population group is considered a pelican, which is an aspirant solution. So, based on each aspirant solution, the objective function of the deployed is analyzed. The value for every objective method is computed using an accurate function vector, shown in Eq. 12.

12 $$\begin{aligned} {\textbf{O}} = \left[ {\begin{array}{c} O_1 \\ \vdots \\ O_a \\ \vdots \\ O_u \\ \end{array}}\right] _{ b \times 1} = \left[ {\begin{array}{c} O(Ri)_1 \\ \vdots \\ O(Ri)_a \\ \vdots \\ O(Ri)_v \\ \end{array}}\right] _{ v \times 1} \end{aligned}$$

Here, *O* is specified as the objective function vector and $T_i$ is defined by the value of the objective function in terms of $i_th$ aspirant solution. Hence, POA provokes the features and strategies of pelicans while trapping and hunting the targeted prey for upgrading the aspirant solutions. This process of hunting and the method utilized in it is assumed in two ways:

1. Moving towards the direction of the prey (investigation phase)
2. Flying on the surface of the water (utilization phase)

#### Moving towards the prey

It determines the position of the pelican’s target prey and advances in the direction of greater altitude. The irregular distribution of the prey is employed to explore and maximize the pelican’s investigative capability. The adjustment to the pelican’s position in each iteration is provided by the Eq. 13.

13 $$\begin{aligned} Ri_{iz}^{i+1}={\left\{ \begin{array}{ll} Ri_{iz}^{s} + ra.\left( l_n-\lambda .Ri_{iz}^s\right) ,T(Ri_t)\le (T(Ri_i))\\ Ri_{iz}^{i} + ra.\left( Ri_{iz}^s- l_n\right) ,T(Ri_t)\le (T(Ri_i)) \end{array}\right. } \end{aligned}$$

Where *t* is specified as the current iteration number, $Ri_{iz}^{s}$ shows the position of $i^{th}$ pelican in the $n^{th}$ dimension; $l_n$ specified as the prey position; $T(Ri_t)$ is the main objective function value; $T(Ri_i)$ shows the fitness function. In the POA framework, a new position for the pelican is obtained when there is an enhancement in the objective function. This kind of advancement is called effective improvement, and the algorithm’s movement towards suboptimal regions has prevented. This procedure is achieved using the following Eq. 14.

14 $$\begin{aligned} Ri_i={\left\{ \begin{array}{ll} Ri_i^s, O_i^s < O_i;\\ Ri_i, \text {else,} \end{array}\right. } \end{aligned}$$

Here, $Ri_i^s$ is denoted as the new status of pelican’s, $O_i^s$ Is the value of the objective function determined by phase 1.

#### Winging on the water surface

The pelican arrives at the water’s surface. It expands its wings towards the water’s surface to catch the fish and gather them into the throat pouch. Thus, this hunting process of the pelican is calculated in Eq. 15.

15 $$\begin{aligned} Ri_{iv}^{p+1}=R_{iz}^s+ \lambda (\frac{V- v}{V}).(2.wx-1).Ri_{iz}^s \end{aligned}$$

Hence, the term *v* is specified as the recent list of iterations; *P* is set as the maximum iteration number; *w* is defined as the surrounding population to explore locally available members to merge an enhanced solution; *wx* represents the random number between (0,1); at this section, the updating has also used to agree or neglect the position of new pelican efficiently, which is evaluated in Eq. 16.

16 $$\begin{aligned} Ri_i={\left\{ \begin{array}{ll} Ri_i^p, O_i^s < O_i;\\ Ri_i, \text {else,} \end{array}\right. } \end{aligned}$$

Here, $Ri^s_i$ is specified as the new status of the $i^{th}$ pelican, and $O^s_i$ sets the objective function value based on phase 2. The pseudocode of the deployed MOD-POA is explained in Algorithm 1.

**Algorithm 1:**
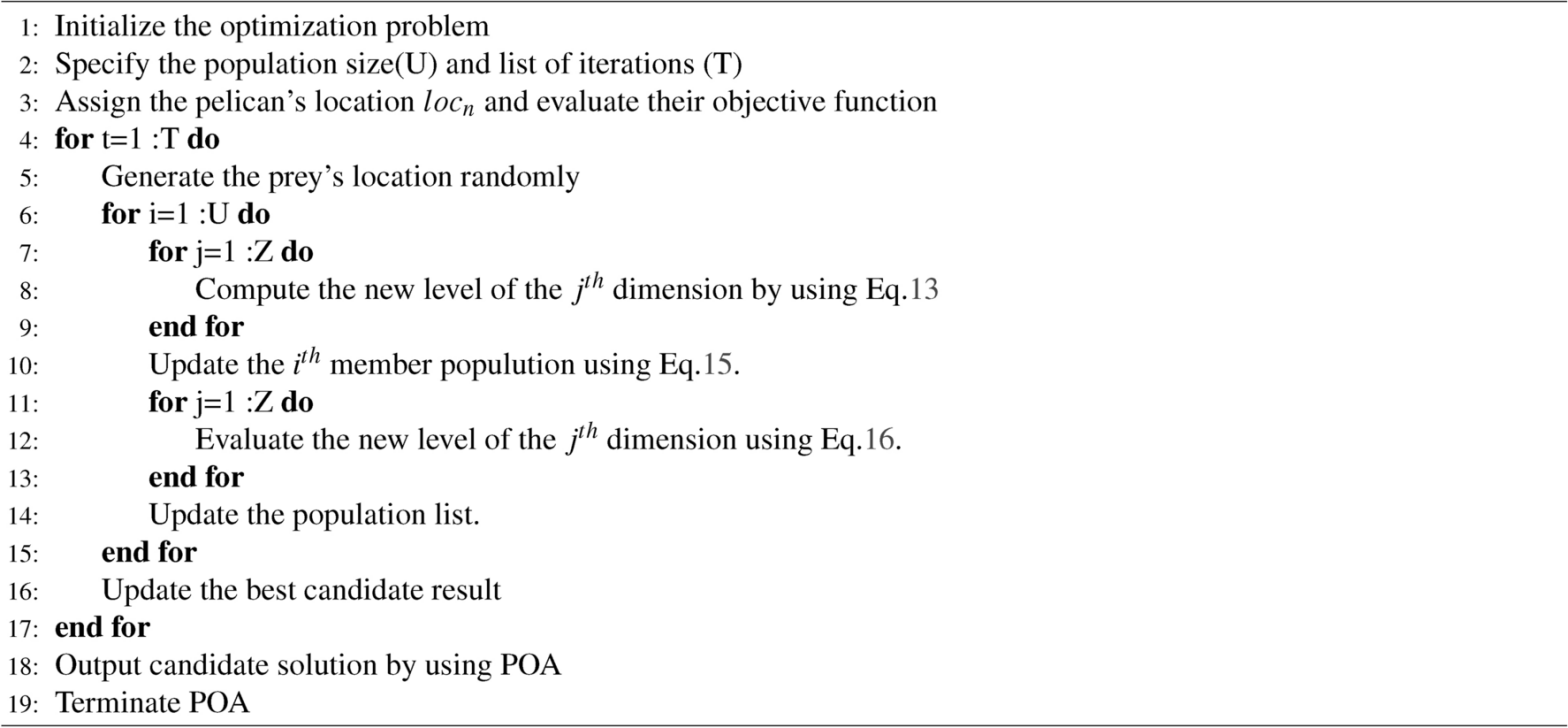
Proposed MOD-Pelican Optimization Algorithm

## Results

The proposed CAD model is run over “PARAM Shavak” supercomputer features an high perperformance computing system incorporates an Intel(R) Xeon(R) Gold 5220R CPU @ 2.20GHz, equipped with either one or two many-core GPU accelerator cards like the NVIDIA P5000 and K40. Additionally, the system includes a minimum of two multi-core CPUs, each possessing at least 12 cores, we have 64 GB of RAM, 8 TB of storage, and three Tera-Flops (TFs) of computing power at their peak outfitted with a development environment for parallel programming and computing power exceeding 2TFs.

In the course of the experiment, we utilized two conventional datasets, namely G1020^52^ and ORIGA^53^ (The link to access the dataset is available in the data availability section). We have resized the images to 128 $\times$ 128. To avoid the problem of over-fitting, we have employed a cross-validation procedure known as FCV. This approach includes dividing the dataset into five separate subsets, with careful attention to avoid any overlap between them. The model is trained in four distinct subgroups, followed by testing in the remaining one. This process is iterated five times, ensuring that each subset is utilized as the evaluation set precisely once. Employing such strategy enhances our ability to gauge the model’s performance while mitigating the risk of overfitting to the training data. This approach splits the entire dataset into training and testing sets used in both datasets.

The used model has been validated on two conventional data sets with images of fundus glaucoma, namely G1020^52^ and ORIGA^53^. The G1020 dataset, which consists of 1020 publicly available retinal fundus images, offers a valuable resource for glaucoma classification, with 724 images representing healthy cases and 296 depicting instances of glaucoma. Furthermore, the Open Retinal Fundus Images for Glaucoma Research and Analysis (ORIGA) dataset stands out as a widely used repository, featuring 650 fundus images. Within the ORIGA dataset, 482 images represent healthy patients, while 168 images are from patients diagnosed with glaucoma.

##  Explainability results

**Fig. 3:**
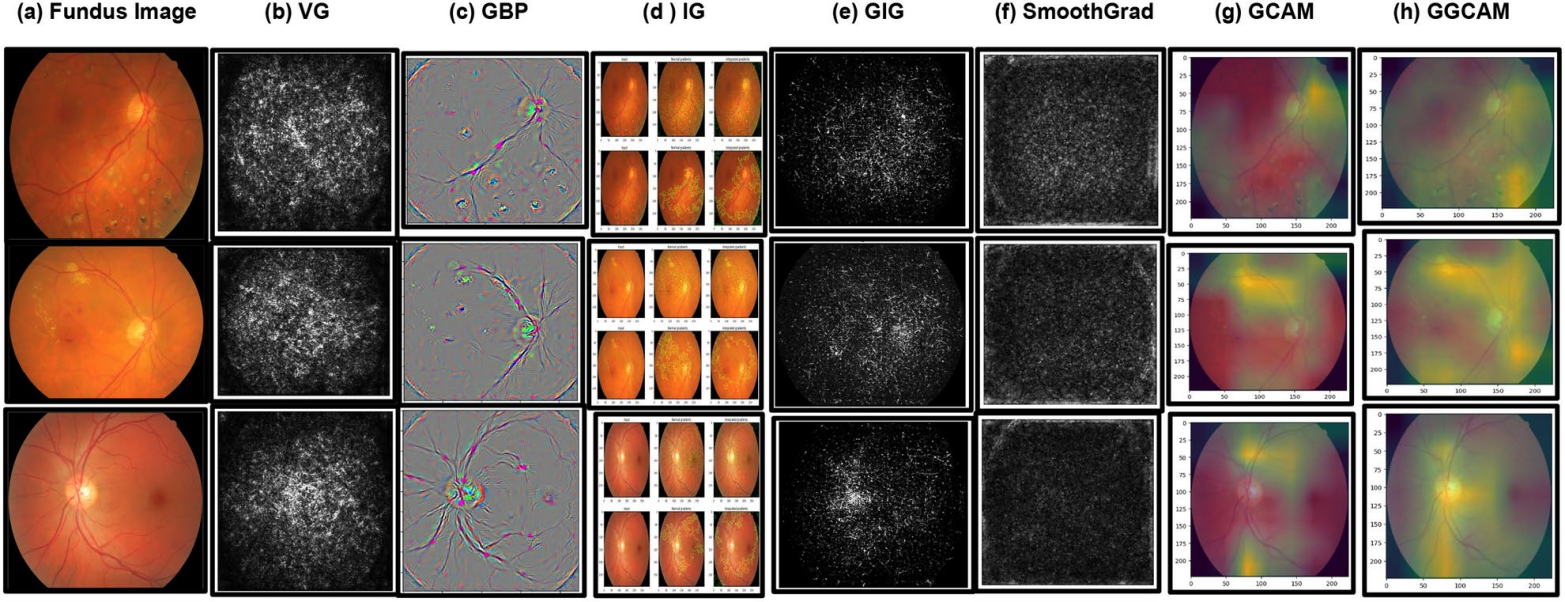
Some sample input images and their respective heatmaps using fundus images datasets like G1020^52^ and ORIGA^53^.

We have implemented the suggested computational framework for Glaucoma classification, and explainability. To enhance the credibility, in order to improve usability and trust, we integrated glaucoma classification and explainability visualizations. The heatmaps produced using list of employed methods emphasize the significant areas on the input image that influenced the predictions. The area for cupping in the middle of the optic disc has closely associated with occurrences of glaucoma^23^. In general, an elevated cup-to-disk ratio (CDR) raises the suspicion and probability of the patient having glaucoma. There are non visible cupping issues and no signs of glaucoma present in the healthy eye. So there are no areas affected by glaucoma to point out and see. The main focus of glaucoma eye is on the optic nerve where the cupping problem is prominent. Usually, the red spots were primarily found at the optic nerve head (ONH), where they have the most significant influence on the categorization. The significance is demonstrated through the highlighted colors in the sequence, starting from the most crucial to the least crucial illustrated in Fig. 3, we contrasted the two interpretability displays using Vanilla Gradients (VG), Guided Backpropagation(GBP), Integrated Gradients (IG), Guided Integrated Gradients (GIG), SmoothGrad, GCAM Gradient-weighted Class Activation Mapping (Grad-CAM), Guided Grad-CAM (GGCAM) methods. In general, combination of overall explainable artificial intelligence (XAI) methods improves by offering superior visual interpretations of MOD-POA+ELM model detection including enhanced object localization, clarification of multiple object instances in one image, and improved explanation-based knowledge transfer compared to Grad-CAM^24^.

The Vanilla gradient (VG) is the simplest way to visualize specific regions in an image that have the most significant influence on the neural network’s classification result^69^, Another method to compute the gradient of a specific output in relation to the input is to employ guided techniques gradient descent is used in reverse to adjust the weights of a neural network through Guided Backpropagation(GBP)^55^. Then, introduced integrated gradients (IG) as a solution for the common problem of saturation came across in methods that rely on gradients^54^. Kapishnikov et al.^56^ introduced guided integrated gradients (GIG) as a method for adoption based on the concept of a path, input image, reference point, and the complex model to be interpreted. Smilkov et al.^57^ proposed a modification to tackle a common issue in gradient-based optimization methods called smoothGrad. In^58^, the authors presented the class activation mapping (CAM) visualization methods for a variety of uses scope of ELM. The GACM employs gradients to offer visual explanations without requiring retraining or improving the design of the model. In order to help advance the development of transparent healthcare automation systems, we integrated XAI methods into our suggested recognition system. In XAI heatmap visualization, significant areas are typically emphasized signaling greater importance for the classification outcome^23^. The strength of the highlighted areas changes based on factors like the model’s prediction certainty and the intricacy of the input image. The key areas of the input are strongly highlighted in red in the sample visualizations of each class in our dataset, showing the model’s certainty. On the other hand, the unimportant regions are emphasized with cool hues, as seen in the peripheral areas of CDR ratio. Implementing this XAI visualization system will benefit HealthCare staff by aiding in both the automated identification of glaucoma categories and in comprehending and confirming machine-generated decisions^25^. Healthcare workers can decrease the rate of decision errors by examining the XAI visualization to comprehend how the intelligent model assigns a particular instance to a specific class^26^. This implies that individuals can have greater confidence in a computer’s assessment of glaucoma detection. Therefore, the glaucoma recognition system powered by XAI will not just automate the identification process, but also instill confidence and dependability in AI system decision-making during diagnosis, resulting in a cost-efficient, effective, and speedy diagnosis system that promotes early medical intervention in severe cases.

## Discussion

**Fig. 4:**
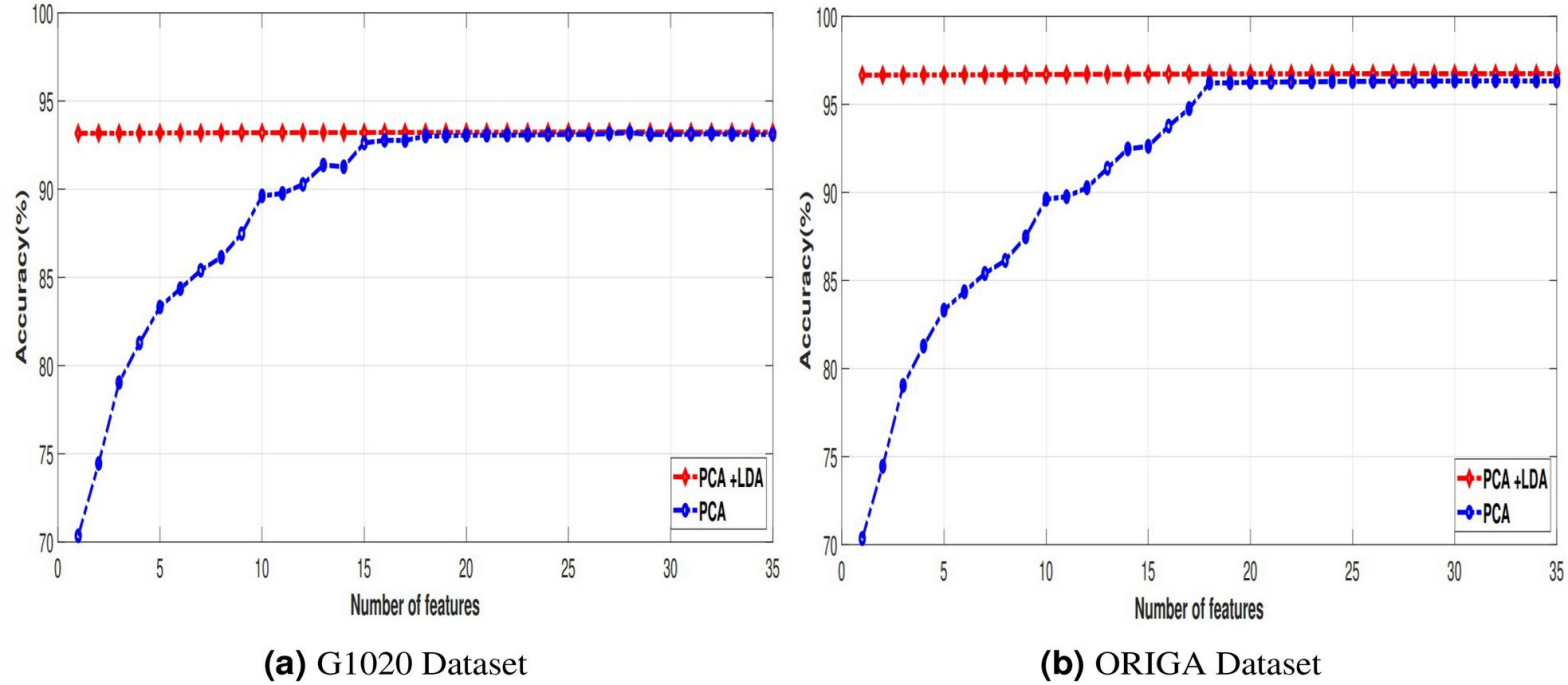
Accuracy examined with respect to the # of features .

**Fig. 5:**
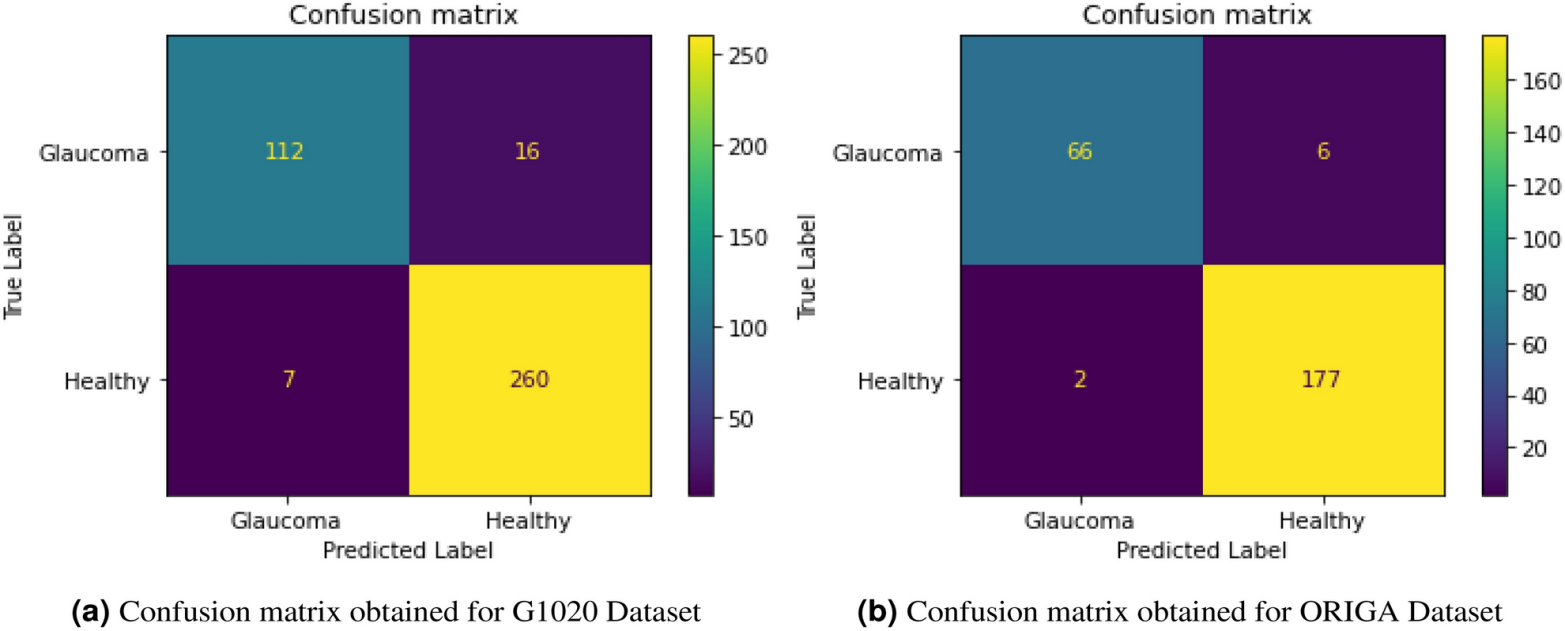
Confusion matrices for G1020 and ORIGA Datasets.

The experimental results of all these schemes have evaluated using two well-established datasets, namely G1020 and ORIGA. We have observed that our proposed MOD-POA+ELM obtains better results with the least hidden nodes over two standard datasets compared with several algorithms namely, KNN, SVM, BPNN, ELM, MOD-ELM, POA-ELM exhibited lower condition numbers and norm values, resulting in improved classification performance. Here, we simulated the novelty of a model deployed with twenty-eighth features. Here, the proposed model is compared with state-of-art models of Various classifiers; Likewise, within the ORIGA dataset, accuracy is observed in algorithms such as KNN, SVM, ELM, MFO-ELM, and POA-ELM as 86.26%, 87.50%, 88.50%, 90.25%, 91.25%, and 92.00% accordingly. Similarly to the ORIGA dataset, the list of algorithms such as KNN, SVM, ELM, MFO-ELM, and POA-ELM heaving accuracy 87.69%, 88.85%, 89.62%, 90.38%, 91.15%, and 92.66% accordingly. The comparison Fig. 4a,b shows that at least 32 features in PCA obtain 93.14%, 96.34% in both G1020 and ORIGA datasets. Then we have implemented combined dimensionality reduction techniques called PCA-LDA has been obtained the best result, that is 93.25% and 96.75% in G1020 and ORIGA accordingly with 28 features described in Table 1. Then, to validate the performance of the proposed model, we employed a 10$\times$5 fold stratified cross-validation (SCV) approach. In each fold, the model is manually tuned with hyper-parameters to learn high-level features. ELM is regarded as the foundational classifier in the classification approach, where the modified POA optimization technique has optimized the weight and biases.We’ve additionally examined our model using an alternative meta-heuristic optimization approach known as MFO^70,71^. In this paper, different conventional classifiers have been utilized, like KNN, SVM, and BPNN. For each classifier mentioned, a population size of 20 and a maximum iteration limit of 100 have been employed in the study. In the experimental results of $10\times 5$ cross-validation with ten iterations shown in Table 2. Convergence of the results of the training sample of one run visualized in Fig. 5a,b.

**Table 1:** Comparative analyses (%) of the deployed model based on PCA versus PCA + LDA method.

Proposed method	No. of features	G1020			No. of features	ORIGA		
		Acc	Sen	Spe		Acc	Sen	Spe
PCA-MOD-POA+ELM	32	93.14	94.78	92.49	32	96.34	97.06	96.07
PCA-LDA-MOD-POA+ELM	28	93.25	93.26	93.22	28	96.75	96.97	96.67

*Acc* Accuracy; *Sen* Sensitivity; *Spe* Specificity

**Table 2:** The effectiveness of the suggested CAD model using the Retinal dataset result(%), 10$\times$ 5-fold, $FO_N$- Fold Number, R-Run, Acc-Average accuracy.

RUN	$FO_N$-1	$FO_N$-2	$FO_N$-3	$FO_N$- 4	$FO_N$-5	Acc
(a) The Retinal G1020 dataset with POA+ELM approaches						
1	91.99	91.99	91.99	90.99	90.99	91.59
2	92.44	92.44	92.44	93.99	90.99	92.46
3	91.99	91.99	91.99	90.99	90.99	91.59
4	92.44	92.44	92.44	93.99	90.99	92.46
5	93.99	93.99	93.99	93.99	90.99	93.39
6	92.44	92.44	92.44	93.99	90.99	92.46
7	93.99	93.99	93.99	93.99	90.99	93.39
8	93.99	93.99	93.99	93.99	90.99	93.39
9	93.99	93.99	93.99	93.99	90.99	93.39
10	91.99	91.99	91.99	90.99	90.99	91.59
(b)The Retinal G1020 dataset with MOD-POA+ELM approaches						
1	91.99	91.99	91.99	91.99	92.99	92.19
2	92.99	92.99	91.99	91.99	91.99	92.39
3	93.94	93.94	93.94	94.66	94.66	94.23
4	91.99	91.99	91.99	91.99	92.99	92.19
5	93.94	93.94	93.94	94.66	94.66	94.23
6	93.44	93.44	93.44	94.66	94.66	94.23
7	93.94	93.94	93.94	94.66	94.66	94.23
8	93.94	93.94	93.94	94.66	94.66	94.23
9	92.99	92.99	91.99	91.99	91.99	92.39
10	91.99	91.99	91.99	91.99	92.99	92.19
(c) The Retinal ORIGA dataset with POA+ELM approaches						
1	95.81	95.81	96.66	96.66	96.66	96.15
2	94.21	94.21	94.21	96.66	96.66	95.19
3	95.81	95.81	96.66	96.66	96.66	96.32
4	95.81	95.81	95.81	96.66	96.66	96.15
5	94.21	94.21	94.21	96.66	96.66	96.19
6	95.81	95.81	95.81	96.66	96.66	96.15
7	95.81	95.81	96.66	96.66	96.66	96.32
8	94.21	94.21	94.21	96.66	96.66	95.19
9	95.81	95.81	95.81	96.66	96.66	96.15
10	94.21	94.21	94.21	96.66	96.66	95.19
(d) The Retinal ORIGA dataset with approaches with MOD-POA+ELM						
1	96.81	96.81	96.66	96.66	96.66	97.72
2	96.66	96.66	96.66	96.81	96.81	99.72
3	96.81	96.81	96.81	96.81	96.81	96.81
4	96.81	96.81	99.66	96.66	96.66	96.72
5	96.81	96.81	96.66	96.66	96.66	96.72
6	96.81	96.81	96.81	96.81	96.81	96.81
7	96.81	96.81	96.66	96.66	96.66	96.72
8	96.66	96.66	96.66	96.81	96.81	96.72
9	96.81	96.81	96.66	96.66	96.66	96.72
10	96.81	96.81	96.81	96.81	96.81	96.81

From the experimental evaluation, we observed that the results obtained from our deployed work demonstrate superior classification performance compared to other existing models while utilizing a reduced number of features. The experimental observations indicate that the proposed MFO-ELM, POA-ELM, and MOD-POA+ELM models achieve lower condition and norm values, resulting in improved accuracy compared to the traditional ELM approach. In Table 3 observation, we have presented a table comparing the performance of different classifiers with our deployed work, named FCDT-WRP-PCA-LDA-MOD-POA+ELM, based on various evaluation metrics like accuracy, sensitivity, and specificity. The results demonstrate that our proposed model compared with two datasets namely ACRIMA and RIM-One performs superior classification in both datasets. The overall explainable methods have been employed to create heat maps for visual explanations. Therefore, a machine learning-based computational model can serve as an effective tool to assist in the identification of glaucoma eye conditions.

**Table 3:** Comparative Analysis (%) deployed CAD scheme Glaucoma datasets with specifications.

Optimization Method + Classifiers	ACRIMA			RIM-One			G1020 Dataset			ORIGA Datase		
	Acc	Sen	Spe	Acc	Sen	Spe	Acc	Sen	Spe	Acc	Sen	Spe
KNN^51^	85.87	83.87	87.42	85.64	79.71	88.89	86.26	81.03	88.11	87.69	86.57	88.08
SVM^51^	86.57	84.68	88.68	86.67	81.16	89.69	87.50	84.75	88.65	88.85	88.06	89.12
RF^51^	87.99	89.31	86.31	86.15	79.71	89.68	87.99	79.66	91.38	88.08	76.12	92.23
XGB^51^	88.69	87.10	89.94	87.50	81.82	90.48	88.24	79.69	91.72	88.46	77.61	92.27
ADB^51^	89.40	87.90	90.57	88.54	83.33	91.27	88.48	81.36	91.79	89.23	79.10	92.75
BPNN^71^	88.61	85.48	88.61	88.02	83.32	90.48	88.50	86.44	89.36	89.62	89.55	89.64
ELM^71^	90.11	88.71	91.19	89.58	84.85	92.06	90.25	90.07	90.68	90.38	90.16	91.04
MFO+ ELM	90.81	89.52	91.82	90.11	86.36	92.06	91.25	90.68	91.49	91.15	90.67	92.54
POA + ELM	91.52	90.32	92.45	91.21	87.88	93.10	92.00	91.53	92.20	92.66	89.55	93.75
MOD-POA+ELM(Proposed Model)	92.23	91.13	93.08	92.31	89.39	93.71	93.25	93.26	93.22	96.75	96.67	96.97

*Acc* Accuracy; *Sen* Sensitivity; *Spe* Specificity

### Performance comparison of existing CAD models

**Fig. 6:**
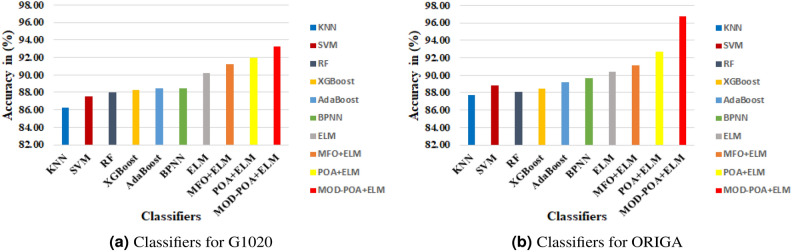
Classification accuracy Datasets.

In this section, the efficiency of the model has been evaluated by comparing it with various advanced classifiers in the field. The evaluation has based on two Fig. 6a,b, allowing for a comprehensive comparison. The overall experiments have been conducted on four publicly available datasets ACRIMA, RIM-One, G1020, and ORIGA obtained the classification results namely 91.45%, 92.43%, 93.25% and 96.75% . The experimental results of the proposed model has been compared and tabulated in Table 4. In our experimental analysis, we observed that our employed scheme compared with explainable methods which achieves superior classification results with both datasets.

**Table 4:** Analysis the classification results with existing models.

Existing methods	Acc(%)				
	Explainability	ACRIMA	RIM-One	G1020	ORIGA
TIA-Net (SOD+Attention)^72^	NA	–	–	–	85.70
2D-FBSE-EWT^73^	NA	–	–	–	91.01
SMOTE + RF^74^	NA	–	–	–	78.30
SMOTE + RF^74^	NA	–	–	–	82.80
HOG +SVM^39^	NA	–	–	83.32	–
HOG + PNN^39^	NA	–	–	87.92	–
HOG + RNN^39^	NA	–	–	85.72	–
SS-SQ-VDM+SVM^18^	NA	92.67	–	–	–
LS-SVM^19^	NA	84.95	–	–	–
QB-VMD^20^	NA	–	86.13	–	–
EWTDWT+LS-SVM^21^	NA	83.57	–	–	–
CVMD +SVM^22^	NA	–	89.18	–	–
CNN^75^	CAM	–	–	–	93.5
CNN^76^	NA	–	90.51	–	–
ResNet-50^77^	NA	–	–	–	92.59
Proposed model	VG, GBP, IG, GIG, SG, GCAM , GGCAM	91.45	92.43	93.25	96.75

*Acc* Accuracy; *VG* Vanilla Gradients; *GBP* Guided Backpropagation; *IG* Integrated Gradients; *GIG* Guided Integrated Gradients; SmoothGrad; Gradient-weighted Class Activation Mapping (GCAM); Guided Grad-CAM (GGCAM)

## Conclusion

In this research, a computer-aided diagnosis (CAD) model has been developed, in which the FDCT-WRP technique has employed to extract curve-like structures from a glaucoma fundus image. This approach effectively captured 2D similarities, such as curves in the images. The dimensionality reduction techniques employed, namely PCA+LDA, have been utilized to identify and select the most relevant features. Hence, POA-ELM methods have followed the merits of enhancing the condition value, which is crucial to achieving improved generalization results and faster learning speed. Hence, the deployed scheme has several limitations. The deployed work has tested and validated using a dataset of glaucoma images, but it has not integrated with multiple image datasets. To focus on two problems, the proposed model is being learned. In addition, we introduce the xAI and its application in medical image classification withe the help of existing work. In future, The task of classifying various classes will be undertaken. Finally, exploring an automated optimization technique with less number of parameters for the proposed MOD-POA algorithm would be beneficial. This would alleviate the need for extensive parameter tuning that is currently required.

## Data Availability

The datasets used and/or analyzed during the current study are available publicly and can be access with the link provide below: G1020: https://www.kaggle.com/datasets/arnavjain1/glaucoma-datasets?select=G1020. ORIGA: https://www.kaggle.com/datasets/arnavjain1/glaucoma-datasets?select=ORIGA. In addition, for dataset specification and its detailed analysis the following can be refer for G1020^52^ and ORIGA^53^.
